# Evaluation of knowledge of Health care professionals on warfarin interactions with drug and herb medicinal in Central Saudi Arabia

**DOI:** 10.12669/pjms.321.8902

**Published:** 2016

**Authors:** Mohamed N. Al-Arifi, Syed Wajid, Nawaf K. Al-Manie, Faisal M. Al-Saker, Salmeen D. Babelgaith, Yousif A. Asiri, Ibrahim Sales

**Affiliations:** 1 Mohamed N. Al-Arifi, Department of Clinical Pharmacy, College of Pharmacy, King Saud University, Saudi Arabia; 2 Syed Wajid, Department of Clinical Pharmacy, College of Pharmacy, King Saud University, Saudi Arabia; 3 Nawaf K. Al-Manie, Department of Clinical Pharmacy, College of Pharmacy, King Saud University, Saudi Arabia; 4 Faisal M. Al-Saker, Department of Clinical Pharmacy, College of Pharmacy, King Saud University, Saudi Arabia; 5 Salmeen D. Babelgaith, Department of Clinical Pharmacy, College of Pharmacy, King Saud University, Saudi Arabia; 6 Yousif A. Asiri, Department of Clinical Pharmacy, College of Pharmacy, King Saud University, Saudi Arabia; 7 Ibrahim Sales, Department of Clinical Pharmacy, College of Pharmacy, King Saud University, Saudi Arabia

**Keywords:** Warfarin–drug interaction, Drug interaction knowledge, Health care professionals, Saudi Arabia

## Abstract

**Objectives:**

To evaluate health care professionals’ knowledge on warfarin interactions with drugs and herbs.

**Methods:**

A self-administered questionnaire was developed to assess health care professionals’ knowledge on warfarin interactions with drug and herb. Respondents were asked to classify 15 drugs that may effect on warfarin action as “enhance”, “inhibit “, “no effect”. The study sample involved health care professionals (physicians, pharmacists and nurses) from king Salman hospital, Saudi Arabia.

**Results:**

About 92.2% of health care professionals identified warfarin interactions with aspirin, 4.4% for warfarin and fluoxetine. Warfarin and cardiac agents (atenolol) was correctly identified by 11.1% of respondents. In warfarin –herb interactions section, the majority of respondents (66.7%) identified the interaction between green tea and warfarin. Approximately one-third of respondents (n=33) correctly classified warfarin interactions with cardamom. No significant difference was found between the health care professionals (p=0.49) for warfarin-drug interactions knowledge score and *p*= 0.52 for warfarin- herb interactions knowledge score.

**Conclusion:**

This study suggests that health care professionals’ knowledge of warfarin- drug-herb interactions was inadequate. Therefore, health care professionals should receive more education programs about drug-drug/herb interactions to provide appropriate patient counseling and optimal therapeutic outcomes.

## INTRODUCTION

Warfarin is the most commonly used oral anticoagulant and has been used in preventing thromboembolic events in patients with chronic arterial fibrillation, prosthetic heart valves, venous thrombosis canonry hear disorders.^1^-^3^ Mode of action of Warfarin believes to exert its effect by lowering the amount of active vitamin K available for the activation of clotting factors II, VII, IX, and X.^4^ Use of warfarin is still limited despite the strong evidence for its clinical value. This may be due to the narrow therapeutic index, warfarin’ drug and herb interactions, and resulting in non-therapeutic anticoagulation or life-threatening hemorrhagic complications.^5^-^7^

In many developing countries, the use of herbal medicines is common among patients with chronic diseases. In Saudi Arabia, prophetic medicine (herbal drugs) is commonly used and practiced by most physicians and the public.^8^ Nearly 70% the public in the central of Saudi Arabia had used practices related to prophetic medicine in their lifetime.^9^

Herbal medicines and food interaction are currently reported as the main cause of adverse events with warfarin. Many publish studies have cited that warfarin interactions with drugs and herbs are common and harmful.^4^,^10^

Healthcare professionals’ recognition of potential drug- drug interactions (DDIs) and herb- drug interactions are essential in reducing the risk of drug-related problem are a serious medical disorders may result in non-therapeutic anticoagulation or life-threatening hemorrhagic complications, and increased cost. Therefore, HCPs in every practice setting need to be alert in monitoring for potential warfarin interactions with drugs and herbs and advising patients regarding herb medicines to avoid when taking warfarin.^5^,^7^,^11^

In Saudi Arabia, no study has been found focusing on the knowledge of the HCPs about warfarin interactions with drug and herb. Hence, we designed the present study to assess HCPs’ knowledge about warfarin interactions with drugs and herbs.

## METHODS

A cross –sectional study was undertaken between March 2014 and June 2014, to examine the HCPs’ knowledge of warfarin- drug and warfarin–herb interactions. The study was approved by research Ethics Committee at College of Pharmacy, King Saud University, Riyadh, Saudi Arabia.

### Study location

This study was carried out at the Tertiary Government Hospital, Riyadh city, Saudi Arabia.

### Study population

The population of this study was the HCPs who are working in Tertiary Government Hospital, Riyadh city, Saudi Arabia. HCPs include all doctors, nurses and pharmacists.

### Sample size

The target population for this study included doctors, pharmacists and nurses working in Tertiary Government Hospital. There were approximately 600 registered HCPs who working in Tertiary Government Hospital. Out of this 150 HCPs (25%) were randomly selected from the register. Stratified random sampling was used in which HCPs were divided into subgroups (i.e. doctors nurses and pharmacists), before selection.

### Survey questionnaire

Items focused on common warfarin – drug interactions^12^,^13^ and other items related to interaction of warfarin with herb 6, 14 were extracted from literature.

The first draft of questionnaire was reviewed by an expert panel which consist of a clinical pharmacist, a pharmacologist, and a physician, all of whom had experience in their respective fields. The questionnaire was pilot tested among eleven HCPs before distribution. The reliability test showed a Cronbach’s alpha of 0.77 for warfarin–drug interactions and 0.51 for warfarin- herb interactions.

The final questionnaire consisted of 22 items. The knowledge of warfarin- drug interactions’ section consisted of 15 multiple choices questions whereas warfarin-herb interactions’ section included 7 questions. HCPs were asked to classify the effect of each drug on warfarin action as “enhance “, “inhibit”, “ no effect” or “don’t know” and asked to identify warfarin interactions with each herb as “yes “, “no” and” don’t know”. Every correct answer in each section was given one mark. The final score in each section was calculated by adding the marks scored in that section.

### Statistical Analysis

The data was keyed into the Statistical Package of Social Science software version 22 (SPSS Inc., Chicago, IL, USA) for analysis. Both descriptive and analytic statistics were applied. For descriptive analysis, results were expressed as numbers, percentages and mean (± SD). The Kruskal-Wallis test was used to assess HCPs group differences at a significance level of 0.05.

## RESULTS

Percentages of correct answers regarding warfarin drug-drug interactions were ranked and the curve of these ranks in each group was not normally distributed. The homogeneity of variance between the three groups (physicians, pharmacists and nurses) was tested using non-parametric Levene’s test. The test showed that the three groups have similar distribution curves (F=1.307, p>0.05). Kruskal-Wallis test showed that there is no significant difference (p>0.05) between the three groups regarding their knowledge of warfarin drug-drug interactions. For drug-herb interactions the three groups were also found to have similar distribution curves (F=0.777, p>0.05) and there was no significant difference between the groups (p>0.05) regarding this type of interactions.

Ninety HCPs returned completed questionnaire (response rate, 56.2%). Out of those 90, there were 24 physician (26.7%), 31 pharmacists (34.4 %) and 35 nurses (38.7%). More than 70% of physicians and pharmacists were male. The majority of respondent had been in their practice for less than ten years (73.3%). More than half of respondents graduated from Saudi Arabia. Table-I shows the distribution of demographic data of health care professionals.

**Table-I:** Distribution of demographic data of health care professionals by groups.

Characteristics	Physician N (%)	Pharmacist N (%)	Nurse N (%)	Total N (%)
*Gender*				
Male	17(70.8)	22(71.0)	14(40)	53(58.9)
Female	7(29.2)	9(29.0)	21(60)	37(41.1)
*Age (years)*				
25-35	9(37.5)	28(90.3)	29(82.9)	66(73.3)
36-45	6(25.0)	2(6.5)	5(14.3)	13(14.4)
46-55	8(3.3)	1(3.2)	1(2.9)	10(11.1)
More than 55	1(4.2)	--	--	1(1.1)
*No. of experiences (years)*				
Less than 10	9(37.5)	26(83.9)	29(82.9)	66(73.3)
11-20	12(50.0)	5(16.1)	5(14.6)	13(14.4)
21-30	2(8.3)	--	1(2.9)	10(11.1)
More than 30	1(4.2)	--	--	1(1.1)
*Country of graduation*				
Saudi Arabia	8(33.3)	28(90.3)	13(37.1)	49(54.4)
Egypt	9(37.5)	1(3.2)	1(2.9)	11(12.2)
Sudan	3(12.5)	1(3.2)	--	4(4.4)
India	1(4.2)	1(3.2)	5(14.3)	7(7.8)
Philippine	2(8.3)	--	15(42.9)	17(18.9)
Bangladesh	1(4.2)	--	1(2.9)	2(2.2)

### Knowledge on warfarin –drug interactions

Correct responses for warfarin- drug interaction ranged from 4.4% (n =4) for warfarin and Fluoxetine may enhance effect on warfarin action to 92.2% (n = 83) for warfarin and aspirin increases likelihood of bleeding. Table-II shows the frequency of respondents who gave correct answers.

**Table-II:** Response of HCPs (pharmacists, doctors, and nurses) to the knowledge questionnaire N=90.

No	Items	Doctors Correct answer N(%)	Pharmacists Correct answer N(%)	Nurses Correct answer N(%)	Total Correct answer N(%)
1	*Anti inflammatory agents*				
	Aspirin	24(100)	26(83.9)	33(94.3)	83(92.2)
	Topical salicyates	16(66.7)	15(48.4)	17(48.6)	48(53.3)
2	*Cardiac agents*				
	Propranolol	17(70.8)	15(48.4)	16(45.7)	48(53.3)
	Atenolol	2(8.3)	4(12.9)	4(11.4)	10(11.1)
3	*Gastrointestinal agents*				
	Omeprazole	10(41.7)	17(54.8)	21(60.0)	48(53.3)
	Ranitidine	4(16.7)	9(29)	1(2.9)	14(15.6)
	Sucralafte	7(29.2)	5(16.1)	13(37.1)	25(27.8)
4	*Antimicrobial agents*				
	Ciprofloxacin	17(70.8)	20(64.5)	15(42.9)	52(57.8)
	Fluconazole	13(54.2)	16(51.6)	14(40.0)	43(47.8)
	Azithromicin	13(54.2)	14(45.2)	16(45.7)	43(47.8)
5	*CNS agents*				
	Fluoxetine	1(4.2)	2(6.5)	1(2.9)	4(4.4)
	Phenytoin	6(25.0)	11(35.5)	11(31.4)	28(31.1)
6	*Vitamin supplements*				
	Multivitamin	3(12.5)	7(22.6)	4(11.4)	14(15.6)
	Vitamin E	5(20.8)	7(22.6)	11(31.4)	23(25.6)
	Vitamin C	12(50.0)	10(32.3)	14(40.0)	36(40.0)

### Knowledge on warfarin–herb interactions

In this section, healthcare professionals were asked to select the most appropriate warfarin interactions with herb. Table-III shows the correct answer of respondents to warfarin-herb interactions knowledge questions. Correct responses for warfarin-herb interactions ranged from 33.3% (n = 30) for warfarin and cranberry to 75.6% (n = 68) for warfarin and garlic.

**Table-III:** Frequency of respondents who gave correct answers about herb interactions with warfarin.

No	Items	Doctors Correct answer N(%)	Pharmacists Correct answer N(%)	Nurses Correct answer N(%)	Total Correct answer N(%)
1	Green tea	17(70.8)	23(74.2)	20(57.1)	60 (66.7)
2	Garlic	22(91.7)	21(67.7)	25(71.4)	68(75.6)
3	Ginkgo biloba	14(58.3)	20(64.5)	13(37.1)	47(52.2)
4	Grapefruit	17(70.8)	22(71.0)	19(54.7)	58(64.4)
5	Cardamom	7(29.2)	12(38.7)	13(37.1)	32(35.6)
6	Cranberry	8(33.3)	10(32.3)	12(34.3)	30(33.3)
7	Ginseng	10(41.7)	16(51.6)	22(62.9)	48(53.3)

### Warfarin-drug/herb interactions knowledge score

The Kruskal-Wallis test was used to test the differences in the knowledge of warfarin- drug interactions and warfarin–herb interactions scores among HCPs groups as shown in Tables-IV. No significant difference was found, however, between the HCPs (p=0.49) for Warfarin-drug interactions knowledge and *p*= 0.52 for warfarin-herb interactions knowledge score.

**Table-IV:** Warfarin-drug/herb interactions knowledge score between health care professional groups.

Items	Professionals category	Mean (median)	P* value
Warfarin- drug interaction knowledge	Doctors	5.6.0 (6.0)	0.49
	Pharmacists	5.7 (5.5)	
	Nurses	5.3 (5.0)	
Herb-Warfarin interaction knowledge	Doctors	3.9(3.5)	0.52
	Pharmacist	4.0(4)	
	Nurses	3.5(3.0)	

* Kruskal-Wallis Test.

## DISCUSSION

Studies on HCPs’ knowledge regarding warfarin -drug /herb interactions are limited. To our knowledge, this is the first study done to evaluate the HCPs’ knowledge about warfarin –drug/herb interactions.

In this study it is clear that HCPs did not recognize all these potentially harmful warfarin- drug interactions. Among anti-inflammatory agents two interacting drugs with warfarin were potentiate the effect of warfarin, only aspirin and warfarin was correctly classified by the majority (92.2%) of HCPs. Warfarin and cardiac agents (propranolol); its moderate recognition rate (53.3%) suggests to explain the prevalence of co- prescription of oral anticoagulants and propranolol that was reported highly probable evidence that it potentiated the effect of warfarin^14^

Another potentiating drug the effect of warfarin was fluconazole; its low recognition (47.8%) resulting in bleeding occur if the anticoagulant dosage is not reduced appropriately.^15^ In addition two drugs inhibiting the effect of warfarin including sucralfate and phenytoin; its low recognition rate (27.8% and 31.3% respectively) of the HCPs. This could lead to reduced anticoagulant response to warfarin.^12^,^16^,^17^ This study also found low recognition rate for atenolol, ranitidine and fluoxetine (11.1%, 15.6% and4.4% respectively). These drugs have no effect on warfarinaction.^12^,^18^,^19^

This study found that the HCPs’ knowledge on warfarin- drug interactions was insufficient. This finding however, varies between HCPs where pharmacists slightly higher knowledge than doctors and nurses although the difference was not significant (*p*=0.49). This could lead to inappropriate patient counseling, change in anticoagulant effect and adverse medical consequences. This finding is consistent with the findings from previous study involving pharmacist, doctors and nurses. A study was conducted by Couris *et.al* 11 among HCPs to assess their knowledge about warfarin-vitamin k drug-nutrient interactions. They found that their knowledge was inadequate in drug-nutrient interactions and general nutrition. Because of different drug pair selection, findings of the present study may not be directly comparable to those of a previous study. However, these studies reported that HCPs’ knowledge of drug- drug interactions was in generally poor.^20^,^21^

Certain herbs are already known and/or potential possibly threatening interactions with warfarin, for example, ginkgo, garlic (Allium sativum), and ginseng (Panax ginseng) potentiate anticoagulant therapy and may alter bleeding times and should, therefore, not be used concomitantly with warfarin.^6^,^14^,^22^

The result of the current study showed that the majority of the HCPs had poor knowledge on herb-warfarin interactions. The HCPs’ knowledge on warfarin and Cranberry interaction was low recognition rate was (33.3%). The other area of weak knowledge among HCPs on warfarin and cardamom interactions 35.6% of HCPs correctly identified. About 52% of HCPs correctly classified the interaction between Ginkgo biloba and warfarin. This is low recognition rate may be resulting inappropriate patient counseling and resulting in major bleeding.^23^,^24^

The finding of present study may not be directly comparable to those a pervious study. Because our study focused only on herb-warfarin interactions while others were among herb- drug interactions. Nevertheless, few common findings are noteworthy. A study was carried by Suchard *et*.*al*^25^ to assess the physician knowledge about herb- drug interaction. The results of this study revealed that the level of physician’ knowledge was poor.

### Limitations

Our study has some limitations. Respondents were from a single hospital of central Saudi Arabia. Therefore, not representative of the entire population of health care professionals in Saudi Arabia as whole. The study did not involve dietitians or doctor of naturopathy, the two HCPs used to provide dietary advice to patients with chronic diseases. This survey can serve as a preliminary study and is helpful in understanding the knowledge of HCPs on warfarin drug- herb interactions in Saudi Arabia..

## CONCLUSION

This study assessed the HCPs’ knowledge on clinically significant warfarin drug - herb interactions. This study suggests that HCPs may have insufficient ability to recognize clinically significant warfarin- drug interactions and herb–warfarin interactions. Inadequate knowledge of warfarin-drug interactions/ herb-warfarin interactions results in insufficient anticoagulation effect or bleeding complications. Therefore, additional training and integration of knowledge and expertise about drug-herb interactions among healthcare professionals is essential to provide appropriate patient counseling and optimal therapeutic outcomes
